# The state of cervical cancer screening in imprisoned women in Malawi: a case of Maula Prison

**DOI:** 10.1186/s12905-023-02349-5

**Published:** 2023-04-28

**Authors:** Regina Mendulo, Isabel Kazanga Chiumia

**Affiliations:** 1 Department of Public Health, Kamuzu University of Health Sciences, Blantyre, Malawi; 2 Department of Health Systems and Policy, Kamuzu University of Health Sciences, Blantyre, Malawi

**Keywords:** Cervical cancer, Prisoners, Screening, Malawi, Sub Saharan Africa

## Abstract

**Background:**

Malawi is one of the countries with the highest burden of cervical cancer in the world with less than ten percent of women screened for cervical cancer annually. The study aimed to investigate the state of cervical cancer screening among incarcerated women at Maula prison. The study highlights key challenges that women in prison face to access cervical cancer screening to inform policies and strategies to address them.

**Methods:**

The study employed a cross-sectional qualitative study design. A total of 31 prisoners aged between 18 to 49 participated in the study. Among these, 15 women participated in in-depth interviews, while 16 women participated in two focus group discussions consisting of 8 women per group. All interviews were recorded and transcribed verbatim. Data was analysed using inductive content analysis.

**Findings:**

Majority of women at Maula prison demonstrated knowledge of cervical cancer, its associated risk factors and the benefits of cervical cancer screening. Most women also expressed willingness to undergo cervical cancer screening. However, the following were identified as factors that hinder women from accessing cervical cancer screening services at the prison:—limited availability of the services, pain during the screening process, the presence of male practitioners conducting screening, poor treatment by authorities and health workers and favouritism.

**Conclusion:**

To improve cervical cancer screening and its uptake at Maula prison there is a need to ensure unlimited availability of the screening services which should be conducted by female health practitioners. There is also a need to include this service as part of the mandatory health screening exercise that is conducted upon entry into the prison by all prisoners. Conducting in-depth awareness and sensitization with participants before screening would help to eradicate fear, provide assurance and clarification of the screening process. Prison officers and health workers should also be sensitised to improve prisoners’ access to healthcare during incarceration.

## Background

Malawi is one of the countries with the highest rate of cervical cancer in the world with an age-standardized rate of 75.9 per 100,000 and human papillomavirus prevalence at 33.6% [1]. It is estimated that 3,684 women develop cervical cancer and 2, 314 die from the disease annually in Malawi [2]. As one of the leading causes of death among women in Malawi, cervical cancer is of primary focus for the Malawi health sector [3]. Public, private for profit, and private not for profit sectors all contribute to the provision of healthcare in Malawi for a population of over 20,000,000 [3, 4]. The four levels of its health system are community, primary, secondary, and tertiary. An established referral system connects these many levels to one another [3]. In the fight against cervical cancer, the Ministry of Health (MoH) in Malawi developed a four-year National Cervical Cancer Control Strategy (2016–2020) that outlines comprehensive interventions to be taken by the government and other partners in mitigating the burden of cervical cancer [3, 5]. The country adopted the single visit screen and treat strategy as part of this Cervical Cancer Control Programme, using visual inspection with acetic acid (VIA) followed by cryotherapy or thermocoagulation at primary health facility level. The current recommendations for VIA screening in Malawi are that women between the ages of 25 and 49 be screened once every three to five years, with yearly Screening for women who have been diagnosed with Human Immunodeficiency Virus (HIV). For women aged 25 to 49 being screened with VIA for the first time within the previous 12 months, a national goal of 80% screening coverage has been set [5]. Cervical cancer screening coverage grew from 9% in 2011 to 26.5% in 2015 across the country [3]. However, it still falls far short of the target with only 34% of eligible women being examined for cervical cancer between July 2020 and June 2021 [4]. While cervical cancer screening services are provided adequately and for free to women by MoH, the situation varies in prisons, even though it is believed that the level of abnormal cervical smear results appears to be higher than normal among this population of women [6, 7].

There were 14,500 prisoners in Malawi’s 30 prisons in 2020 [8]. Of these prisoners, female prisoners accounted for 1.1% of this population [8, 9]. According to the Inspectorate of prisons, the Malawi government remained largely noncompliant with the High Court’s requirement to improve prison conditions [10]. Detainees’ rights to access specific medical care depend upon the availability of the service to the general female public within a given country. This position is reflected in Rule 10 of the United Nations Rules for the Treatment of Women Prisoners and Non-custodial Measures for Women Offenders (Bangkok Rules of 2010) which states that gender-specific health care services in prisons must at least be equivalent to those available in the community [11]. Section 42 (1, b) of the constitution of the republic of Malawi provides for the right to be detained under conditions consistent with human dignity, which shall include at least the provision of reading and writing materials, adequate nutrition, and medical treatment at the expense of the State [12]. Although the legal framework safeguards the rights of prisoners, the prison conditions do not conform to the standards set by the constitution or instruments of international law of which Malawi is part of [13]. It is to this view that the study sought to investigate the state of cervical cancer screening among women during incarceration. This study is based on the premise that there is inadequate understanding of female criminology and that the confinement of female prisoners raises several healthcare difficulties for the prisoners. The study highlights key challenges that women in prison face to access cervical cancer screening to inform policies and strategies to address them.

## Methods

### Study design, target population and study period

The study selected Maula prison due to its reputation as one of the largest and most overcrowded prisons in the country, providing a valuable opportunity to examine the conditions and challenges faced by the prison system. It employed a cross-sectional qualitative study design to obtain an in-depth understanding of the situation and an overall picture at the time of the study [14]. The study targeted all female prisoners at Maula prison that were above 18 years of age and within the reproductive age group (18–49 years) who were either sentenced or on remand. The study was conducted from January 2018 to August 2020. Data was collected from February 2020, analysed and compiled in May 2020 followed by report writing in August 2020.

### Inclusion criteria

The inclusion criteria included women between 18 to 49 years of age who were at an increased risk of developing cervical cancer, had at least attended primary school education, with or without screening history and women who were imprisoned at Maula prison in Malawi; on remand (detained awaiting sentencing) or were serving their sentence.

### Exclusion criteria

The study did not target any women over 49 years of age, pregnant women and women who have declined to participate in the study or were unable to provide informed consent.

The table below describes the population Table 1.

**Table 1:** Characteristics of population of interest

Characteristics	Description	Total Population	Target population	Sample Population	IDI sample	FGD sample
Marital Status	Married	40	22	19	10	9
	Single	35	15	9	3	6
	Other- Divorced	9	3	3	2	1
Age	18–25	42	18	9	4	5
	26–49	28	22	14	6	8
	49 +	14	0	8	5	3
Number of Children	None	20	10	6	2	4
	1 to 2	36	20	15	8	7
	3 +	28	10	10	5	5
Education background	No education	11	0	0	0	0
	Primary School	40	22	19	9	10
	Secondary school	30	15	9	5	4
	Tertiary education	3	3	3	1	2
Prison sentence	1 -6 months	20	7	5	3	2
	7–18 months	44	18	15	7	8
	19 +	20	15	11	5	6
Screening History	Screened before	45	25	20	8	12
	Never screened	39	15	11	7	4

### Sample selection

The prison wardens were available throughout the study, monitoring and supervising the prisoners as mandated. During the entry meeting, the wardens, clinic officers and the Officer in Charge were briefed on the purpose and inclusion criteria of the study to effectively support the identification of the target group since prisoners live in confined areas with limited access by the researcher. The researcher did not establish any relationships with the study participants prior to the study, however, the researcher conducted introductions and briefed participants of the study aim and goal. The participants were informed by the researcher about her interest in the prison population, which stemmed from a previous job working on a prison health project.

There was a total of 84 female prisoners as of 22 February 2020 at Maula prison. Out of 84 prisoners, 40 were willing to participate in the study while 31 met the inclusion criteria. A total of 15 participants were purposively selected to participate in in-depth interviews (IDI) based on their age (≥ 18 to 49 years), voluntariness, knowledge of cervical cancer and their educational background to differentiate the group’s level of understanding of cervical cancer. Additionally, 16 women were selected to participate in focus group discussions (FGDs). The participants were divided into 2 groups of 8 participants in each group, using convenient sampling [15]. Thus, in total the whole qualitative sample constituted 31 participants and was based on data saturation whereby the information collected became repetitive and the collection of new data did not shed any further light on the issue under investigation [16].

### Data collection

The study used interview guides to collect data through IDIs and FGDs. The tools consisted of questions including the socio-demographic profile, knowledge of cervical cancer, access to screening services, prison conditions in relation to health, benefits of screening and challenges faced in the prison in accessing health care and screening. Interview guides were developed in English and translated into the local language, Chichewa for easy communication and interaction. Data collection tools were pilot tested to ensure reliability and consistency. There were no repeat interviews carried out and no transcripts were returned to participants for corrections. All interviews were carried out face to face and lasted 45 min for each IDI and 60 min for each FGD.

### Data management and analysis

All interviews and FGD were audio recorded and transcribed verbatim using Express Scribe Transcription software. Manual transcription was also done to ensure all the information was captured accurately. To enhance credibility, field notes were captured during data collection as well as triangulation discussions among researchers. Data analysis was conducted using inductive content analysis approach. Inductive content analysis is a method for identifying, analysing and reporting patterns or themes within the data [17]. Systematic coding was done and a code book in Microsoft Excel was used to organize and manage the data. Two researchers independently coded separate transcripts to establish reliability and validity of coding categories, and any differences were resolved through iterative discussions between them. These were then validated by both researchers. The analysis was done in five stages. The first stage involved going through all the transcripts to get familiar with the data. During this stage, short notes were made on transcripts and highlighter colours were used to indicate keywords or statements within the text. The second stage involved eliciting codes from text line-by-line to generate initial codes. The third stage involved sorting and categorical grouping of codes into sub-themes. The fourth stage involved theme generation through the merging of sub-themes. The last stage involved producing the analysis report. Study participants did not provide any feedback to the findings.

## Results

The following were the characteristics of the participants for the study, women aged between 18 and 35 years of age with the majority as married with children. Half the participants had completed their primary school education before incarceration. The participants reported their sentences ranging from 1 to 36 months in incarceration. The study recorded 1 participant to be HIV positive and 1 participant to have had cervical cancer. The study did not record any reasons for the imprisonment of its participants, because the characteristic was not contributing to any of the study objectives and therefore would not have added any value to the study findings.

### Knowledge of cervical cancer and its associated risk factors

The majority of the women in both in-depth interviews and FGDs reported having heard about cervical cancer at least more than once. The most commonly reported source of knowledge was from attending Primary school education and from media awareness messages that were heard over the radio and television. Some women described cervical cancer as;

*“A disease where a woman is found to have sores around the cervix and is caused by having sexual intercourse from a very young age.”* (IDI Participant 8, 3 months incarceration)

*“This cancer disease is worse than HIV because when you are found to be HIV positive, you can still live a long full life with the ARVs which are provided by the government for free, but for cancer, I have heard that you have to go to India to get treatment, otherwise here in Malawi, you will die.”* (FGD1 participant 3, 5 months incarceration)

Upon exploring the possible risk factors associated with cervical cancer, the women reported the following risk factors:—use of vaginal muscle tightening chemicals and soaps, reoccurring vagina infections, having multiple sexual partners, and use of contraceptives.

*“You know women talk and share tips about making a man feel good when they are in bed with you. One of my friends was putting muscle-tightening chemicals and soaps in the vagina to keep it small for men. She got very sick. I believe It is those medicines that can also cause cervical cancer.”* (FGD 2, Participant 8, 8 months incarceration)

While over half of the participants agreed that inserting chemicals was a possible contributing factor to cervical cancer, some of the women believed that cervical cancer is mainly caused by a general lack of personal hygiene of genitals.

### Access to cervical cancer screening services at Maula prison

During the interaction with the Officer in Charge of the Prison to assess the environment of the female prison in terms of health care provision and the availability of screening services, it was discovered that Maula prison had a total of 84 female prisoners in incarceration, a functional dispensary, and a consultation room. It was also reported that cervical cancer screening services were made available in the prison randomly depending on the availability of the screening entity (both government and non-governmental organisations). When asked about the willingness of prisoners to participate in the screening exercises, it was reported that there was good representation with more women agreeing to screen than those that did not agree to. Similarly, interaction with the prisoners also revealed that Cervical cancer screening services are not readily available at the prison clinic and they are outsourced just as described by the Officer in Charge. Women who show cervical cancer-like symptoms at the prison clinic are referred to Kamuzu Central Hospital for screening and treatment.

*“Screening is only available when organizations come to do the screening. It is not available at the prison clinic. And it is difficult to go for screening while we are here, in prison.”* (FGD1, Participant 3, 5 months incarceration)

*“There was a time I was suspecting that I have cancer of the cervix. I was bleeding but yet I have already reached menopause. I was also experiencing sharp back and abdominal pain. This was during the time I was already here in prison. I reported to the clinic to request for screening, the clinic was unable to do so, but they arranged for me to go for a check-up at Central hospital.”* (IDI participant 7, 10 months incarceration)

### Perception of prisoner’s health status

The study explored women’s perception of their health status before and while in prison, the different circumstances that women face in the prison and how that affects their sexual reproductive health. Results show that most inmates believed their health status was declining during the period of incarceration as compared to the time they were not in prison. They cited poor diet and hygiene, limited access to health services, extended length of time to process hospital referral requests and pro-longed scheduled visits for some medical procedures, as some of the factors affecting their health. Some prisoners expressed interest to go to private hospitals for better treatment, but that is not allowed even if the prisoner and their families are willing to meet the cost. Hospital referrals in the prison usually take time to process and are made only to government hospitals.

*“It takes a minimum of 7 days to process a hospital referral. Unless it’s an emergency, the internal processes to finalize a referral take long*. *The issue is presented to the officer on duty and the officer has to contact the Police who were responsible for bringing me to the prison, after that then they arrange for me to go to the hospital. By the time they come to get me to the hospital, I have suffered for a long time.”* (FGD2, Participant 7, 24 months incarceration)

Other than ordinary consultations, some medical services have specific dates to be provided to the prison population and not basing on demand or need. These include cancer screening services, HIV and Tuberculosis testing and treatment among others. Periodic provision of these services contributes to an unhealthy prison population altogether.

*“You might have come into prison with the disease and because there is no good screening here, the problem may be worsening.”* (FGD1 participant 4, 24 months incarceration)

*“I have been diagnosed with cervical cancer, and I had scheduled visits to review my health. While I am here, I have no idea if the situation is worsening or the same since we are not allowed to go out for check-up visits.”* (FGD2 participant 5, 36 months incarceration)

Other than the environment, the prison dietary conditions were also perceived to affect women’s health while in prison, especially women with pre-existing conditions such as HIV.

*“for me who is HIV positive, while I was at home, I made sure I ate food and fruits that would boost my immune system and complement my diet, while here in prison, we have no choice but to eat what is provided for us and eating fruits is considered a luxury here in prison.”* (IDI participant 5, 24 months incarceration).

### Benefits of cervical cancer screening

The participants reported early diagnosis and early treatment for the prevention of cervical cancer as some of the benefits of screening. Some women explained during the FGDs that going for screening was good in order to know their status and be assisted accordingly.

*“when they catch cancer at an early stage, it can be treated before it spreads to the other parts of the body. I lost my uterus after giving birth to my firstborn, lucky enough they found that I had cancer while it was still early and now, I am fine.”* (IDI participant 8, 3 months incarceration)

*“It is also good to do the screening, especially for young girls who are about to start having families, it’s good for their planning.”* (FGD2 participant 5, 36 months incarceration)

The participants further explained that they were more willing to go for screening when they were advised by medical doctors to do so. They believe if the advice was coming from the medical doctor, then there could be significant reasons why meaning they may be carrying the signs and symptoms of the disease. On the other hand, some women described their willingness to undergo screening after a friend had recommended it and has undergone screening with a negative outcome (no cervical cancer). They continued to say the topic was less fearsome when someone they knew assured them.

*“When I was growing up, my mother told me there are two people who I must never lie to, a doctor and a teacher. So, if a doctor says I need screening, I cannot refuse or hide my condition. Those people know a lot about the body.”* (IDI participant 10, 18 months incarceration)

### Challenges faced by inmates in accessing cervical cancer screening services

The study explored some challenges that women face when accessing cervical cancer screening services and how this affects their willingness to go for screening in the prison. There were several factors including, pain during the screening process, the presence of male practitioners conducting screening, poor treatment by authorities and health workers and favoritism.

### Pain during the process

Half of the women in one of the FGDs mentioned that screening was a painful process. The extent of the pain varied among the participants, from extreme pain to a mere discomfort. The women perceived that contracted muscles of the vagina wall due to sexual inactivity and use of cold water when cleaning genitals was the cause of the pain. Additionally, continuous bleeding for 48 h after undergoing screening and elongated menstruation with heavy bleeding following screening was another issue’s participants experienced and shared after undergoing screening. These issues negatively affected their life in prison since sanitary pads in the prison were scarce.

*“after I came back from screening, I started bleeding continuously for a week, this happened while I had already had my menstruation the previous week.”* (IDI participant 1, 28 months incarceration)

### Presence of male practitioners conducting screening

Some of the women reported that they felt uncomfortable to undress and undergo the procedure with male health service providers. The women therefore opted to shun away from undergoing screening when it’s conducted by men. The women described the matter as embarrassing and scary because it’s easy to get raped.

*“It is easy to undress in front of another female and it is also less scary because women don’t rape each other.”* FGD1.

*“It is embarrassing for a man to see my private parts after a long time.”* (IDI Participant 15, 14 months incarceration).

### Poor treatment by authorities and health workers

Additionally, the study also revealed that despite the availability of the screening services from time to time, one of the barriers to accessing screening services was due to poor treatment of the prison authorities and health workers providing the services. The treatment instils fear in the inmates, and they tend to look away from seeking care. Most of the women believed that prison authorities and the medical personnel in the prison feel that prisoners are bad people who have lost their rights as long as they are serving their sentence.

*“The officer was harsh and shouted at me during the screening process. The equipment they use is very good, but it is painful when it is inserted. I was not allowed to express any pain or discomfort during the process because I am a prisoner.”* (IDI participant11, 24 months incarceration)

### Favouritism

For most women at the prison, access to cervical cancer screening services was not easy, not all inmates are screened and informed of the outcome of the screening, only a selected few have this access to the services due to favouritisms by prison officers. While most of the long-sentenced prisoners (more than 24 months incarceration) described the access of the service as free to the public (for all who wish to access in the prison), short- sentenced inmates had a different view of this. The study revealed that inmates that have been in the prison longer tend to gain favours from the prison authorities and benefit from interventions that come to the prison most often unlike the others.

*“Sometimes we are forced to participate in events that we don’t want to and other times, we are refused to participate in events that we were willing to take part in.”* (IDI participant 9, 10 months incarceration)

*“I was told I have the virus for cervical cancer a year after the time I was screened.”* (IDI participant 12, 26 months incarceration).

## Discussion

While lack of knowledge of screening programmes is the most common reason for not being screened for cervical cancer among women in the society [17, 18]  the situation is different among imprisoned women at Maula Prison. In this study, lack of information did not come out as a hindrance to screening at all. Knowledge of the disease was universal, while some of the women were well-informed about its source as a sexually transmitted virus, others reported misconceptions and myths about the disease. The participants in this study perceived cervical cancer as dangerous, common and may affect any woman of childbearing age. Earlier studies, from Malawi suggest trends of increasing knowledge about cervical cancer and a heightened sense of susceptibility [19]. The Malawi National Cervical Cancer Control Strategy of 2016–2020 outlines comprehensive interventions to be taken by government and other partners in mitigating the burden of cervical cancer such as awareness of the disease through mass media across the country, this may have contributed to the increase in knowledge of cervical cancer among others [3, 6].

Despite having most of the participants undergone screening once or never, most of the women understood the benefits of screening and diagnosis at an early stage of which they believed avoids adverse effects of the disease. The main barrier that affected the women’s access to screening while in prison was the inconsistency of the services. Recent studies in Malawi have reported high knowledge of cervical cancer against low screening among women in Malawi [19] One of the studies in Malawi discovered high awareness of cervical cancer with about half of the participants as screening-inexperienced (never screened or screened for the first time) while all of the women understood the benefits of screening and the importance of early identification before the cancer progresses [19].

Poor diet and hygiene prolonged scheduled visits and poor referral system were contributing factors to poor women’s health in the prison. The women perceived being in prison has a negative impact on their health. Unsanitary conditions of the prison made the health conditions worse for the women as they often resulted into recurrent infections and transmission of communicable diseases [20]. It is of this view that the government of Malawi must consider and look beyond correction purposes of prisons and begin to focus on wellbeing of its prisoners since health of the prisoners [21].

Despite having knowledge and willingness to undergo screening, the women expressed concern over favouritism and poor treatment by prison officers and health care givers as a barrier contributing to low screening. This was described as demotivating and demeaning for the women. It’s believed that the poor treatment of inmates was as a result of increasing pressure on the human resource to manage the increasing numbers of inmates at the prison. A 2018 Malawi Inspectorate of Prisons Report to the Malawi Parliament indicated to have 630% over capacity with a total of 3026 inmates against 480 recommended capacity of the Maula Prison and an overall 260% over capacity in Malawi prisons, with 14,778 prisoners occupying spaces built for only 5,680 persons [22]. With the prisons at overcapacity, workload of prison officers may be overwhelming which may result to poor service provision and treatment to the inmates. This therefore calls for mindset change interventions by channelling information about prisoner’s health to prison authorities with the aim of improving their attitudes and in return improve the prisoners’ access to health care.

It was clear from the participants that screening was essential in preventing Cervical Cancer, however, the findings of this study revealed that the participants experienced and perceived cervical cancer screening as unpleasant, uncomfortable and painful resulting into refraining from the exercise [23]. This finding explained why there is good knowledge of the disease with low screening levels. Misconceptions of the screening processes that the women shared among themselves in the prison affected their participation in screening. Similarly, Women who received negative results from screening previously, were likely to recommend screening to others and it was highlighted that confidence and relief was a guarantee after undergoing screening particularly after a negative result. This therefore, emphasised the importance of interpersonal relationships for promoting cervical cancer screening [19, 24, 25]. The study participants that underwent screening before incarceration disclosed to have known someone who was diagnosed of cervical cancer as one of the reasons they underwent screening [23].

Preference of having female practitioners conducting the screening exercise other than male health practitioners was another barrier to screening in the prison. The participants described the presence of a male practitioner as a hindrance to women’s willingness to access screening. Due to the nature and sensitivity of the exercise, the women reported to have felt shy and uncomfortable to undress in the presence of a male unlike fellow women. In the attempt to increase the screening culture among women prisoners, use of female health practitioners may significantly impact the practice [24].

Incarcerated women generally experience gender-specific health-related challenges, which include menstruation, pregnancy and childbirth, care of their children within and outside of prison, development of certain forms of cancer, and are often exposed to gender-based violence in the form of physical/sexual abuse by prison officers and male prisoners [26]. The Maula prison was not exceptional over these conditions. In light to the general sexual reproductive health of the women prisoners, toilets and bathrooms used in the prison were not to the best hygienic and sanitary condition. There was inadequate provision of disinfectants for regular cleaning of floors, utensils and toilets. It was discovered during the study that disinfectants are usually on low supply or at all not available for months in the prison with insufficient, overflowing, non-functional toilets and bathing facilities with some water points close to sanitation outflows, and bathing buckets sometimes used as toilet facilities in the night [27]. This was assumed to increase the spread of infectious diseases among the women prisoners. Furthermore, the medication to treat infections such as Candidiasis among others, are also inadequate and often not available at Maula Prison. This therefore indicates that cases of disease infections may be persistent, reoccurring and usually untreated among the prisoners. This finding is in line with a study that conducted a systematic review of dynamic models of infectious disease transmission in prisons and the general population. In this study, it was perceived that Incarcerated populations experience elevated burdens of infectious diseases, which are exacerbated by limited access to prevention measures. The study revealed that prison-based screening and treatment may be highly effective strategies for reducing the burden of HIV, HCV, and other sexually transmissible infections among prisoners [28].

### Study limitations

The study did not interview prison clinic officers to discuss their views of the matter due to limited human and financial resources. Furthermore, the study did not review prison clinic records to establish the level of participation and frequency of availability of cervical cancer screening exercises at the prison. To ensure representation of findings at national level, future similar studies should apply mixed methods approaches with larger samples. These future studies are also needed to establish the factors that affect cervical cancer screening in imprisoned women from the prison management’s perspective. This literature may contribute to policy and intervention approaches for prison health. However, the strengths of the study dwell on the thoroughness of the data collection and analysis for the women prisoners. The methods used in obtaining data support and provide insights into the qualitative aspect of cervical cancer screening in imprisoned women at Maula prison.

## Conclusion

The study revealed that the imprisoned women at Maula prison had knowledge of cervical cancer and its associated risk factors even though some women reported misconceptions. The women in the prison perceived their health was deteriorating during the period of incarceration as a result of poor prison conditions and environment. The women expressed interest to undergo screening in the prison with a few challenges described as a hindrance to the screening process. Recommendations to improve cervical cancer screening and its uptake at Maula prison include the need to ensure unlimited availability of cervical cancer screening services which should be conducted by female health practitioners. There is also a need to include this service as part of the mandatory health screening exercise that is conducted upon entry into the prison by all prisoners. Conducting in-depth awareness and sensitization with participants before screening would help to eradicate fear, provide assurance and clarification of the screening process in the prison. Prison officers and health workers should also be sensitised to improve prisoners’ access to healthcare during incarceration.

## Data Availability

Data and materials supporting the conclusions used in the manuscript are available from the corresponding author on request.

## Abbreviations

FGD: Focus group discussion
HIV: Human immunodeficiency virus
IDI: In depth interview
MoH: Ministry of health
VIA: Visual inspection with acetic acid
